# Primary tumor microRNA signature predicts recurrence and survival in patients with locally advanced esophageal adenocarcinoma

**DOI:** 10.18632/oncotarget.12832

**Published:** 2016-10-24

**Authors:** Daisuke Matsui, Ali H. Zaidi, Samantha A. Martin, Ashten N. Omstead, Juliann E. Kosovec, Luai Huleihel, Lindsey T. Saldin, Christina DiCarlo, Jan F. Silverman, Toshitaka Hoppo, Gene G. Finley, Stephen F. Badylak, Ronan J. Kelly, Blair A. Jobe

**Affiliations:** ^1^ Esophageal and Lung Institute, Allegheny Health Network, Pittsburgh, PA, USA; ^2^ McGowan Institute for Regenerative Medicine, University of Pittsburgh, Pittsburgh, PA, USA; ^3^ Department of Pathology and Laboratory Medicine, Allegheny Health Network, Pittsburgh, PA, USA; ^4^ Department of Medicine, Division of Hematology and Oncology, Allegheny Health Network, Pittsburgh, PA, USA; ^5^ Sidney Kimmel Comprehensive Cancer Center, Johns Hopkins University, Baltimore, MD, USA

**Keywords:** esophageal adenocarcinoma, prognostic marker, miRNA, miR-652-5p, miR-7-2-3p

## Abstract

Esophageal adenocarcinoma (EAC) is an aggressive cancer necessitating the development of improved risk stratification tools for personalized care. Previously, microRNAs have been shown to correlate with the progression and prognosis of various cancer types; however, the value in EAC remains largely unexplored. We performed global microRNA profiling on 32 formalin-fixed, paraffin-embedded EAC specimens to identify microRNAs associated with progression. Literature search and pathway analysis further refined output to five significantly deregulated candidate biomarkers. Four of the five microRNAs (miR-652-5p, miR-7-2-3p, miR-3925-3p, and miR-219-3p) were validated by qRT-PCR. Survival outcomes were evaluated in testing set of 26 stage II/III EAC patients to determine the prognostic relevance of the selected microRNAs. In the testing set, miR-652-5p and miR-7-2-3p expressions were significantly associated with progression-free survival (p-value = .00771 and p-value = .00293). The highest area under receiver operating characteristic (ROC) curve was 0.8212 for the combination of miR-652-5p and miR-7-2-3p. Collectively, our findings demonstrated that the miR-652-5p/miR-7-2-3p signature may serve as a promising prognostic marker in patients with locally advanced EAC.

## INTRODUCTION

Esophageal cancer is the eighth most common cancer and the sixth most lethal cancer worldwide [1]. Esophageal cancer is composed of two main histological subtypes; esophageal squamous cell carcinoma (ESCC) and esophageal adenocarcinoma (EAC) [2]. Most strikingly over the past four decades, there has been an exponential increase in the incidence of EAC in the western hemisphere [3, 4]. Despite advances in the surgical, radiotherapeutic and chemotherapeutic management of EAC, afflicted patients have a poor prognosis with an overall 5-year survival rate less than 20% [5, 6]. Although the tumor node metastasis (TNM) staging system may inform prognosis and therapeutic approach, stage-matched EAC patients can have considerable variation in survival, despite receiving identical therapy [7]. Clearly, it is critical to identify the unique molecular mechanisms underlying EAC development and progression for improved patient staging and the development of novel targeted therapy options.

MicroRNAs (miRNAs) are short non-coding RNAs of 20-24 nucleotides that bind to the 3’-untranslated region (UTR) of corresponding target mRNA to post-transcriptionally repress translation [8]. MiRNAs significantly influence numerous cancer-relevant processes such as differentiation, proliferation, migration, and apoptosis by targeting developmental pathways [9, 10]. Furthermore, accumulating studies have shown that aberrant miRNA expression is involved in the initiation and development of various types of cancers, including esophageal cancer [11–13]. Previously, lymph node involvement and decreased survival in ESCC patients has been shown to be linked to overexpression of miR-21 [14]. Liu et al, have demonstrated that silencing of miR-143 and miR-145 are linked with risk of development of esophageal cancer [15]. Hence, the use of miRNAs as prognostic biomarkers has become increasingly relevant with the potential goal of guiding personalized treatment strategies, especially since the current conventional prognostic factors are inadequate.

Additionally, studies establishing the utility of miRNA signatures in the management of EAC have focused on identifying a unique miRNA expression to distinguish EAC from Barrett's esophagus (BE) and normal squamous epithelium, in order to identify high-risk patients for progressing to EAC [16–18]. However, the miRNA-mediated mechanisms underlying tumor progression and metastasis in EAC are not fully understood. Identifying patients with the highest-risk for developing metastasis would be extremely useful in guiding treatment with the ultimate goal of improved survival. Therefore, development of novel miRNA biomarkers in EAC patients may inform future treatment decisions for patients with regard to the severity of disease, and the aggressiveness and/or effectiveness of the therapy. The present study presents a unique miRNA expression profile as a prognostic tool relevant to EAC progression using comparative miRNA analysis.

## RESULTS

### miRNome analysis

The comparative miRNome analysis identified 80 miRNAs which were significantly down-regulated with greater than 4-fold change in Stage I Progressors compared to Stage I Non-Progressors.

**Figure 1:**
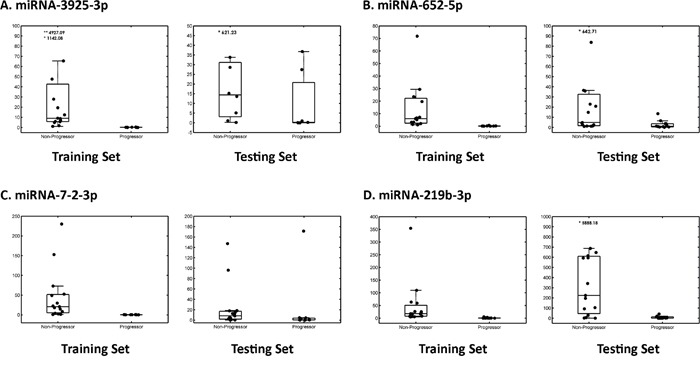
Box plot of four candidate miRNAs in the training and testing set Box plots illustrate the distribution and abundance of candidate miRNAs in tissue samples from patients with EAC Progressors versus EAC Non-Progressors using a qRT-PCR platform. Scatter plots are overlaid on top of the box plots to visualize the individual data points for **A.** miR-3925-3p, **B.** miR-652-5p, **C.** miR-7-2-3p and **D.** miR-219b-3p, respectively. For each candidate miRNA, the left presents results from the training set, and the right box presents results from the testing set. The bottom and top horizontal lines delineating each box plot indicate the first and third quartiles of the data, respectively, and the horizontal line inside each box plot indicates the median value. The length of the box plot whiskers is specified as 1.5 times the interquartile range (25th to 75th quartiles) of the data.

### Literature search and pathway analysis

The literature search identified 12 of 80 miRNAs were linked to carcinogenesis. Downstream analysis of the predicted target genes and pathways of the 12 candidate miRNAs identified 20 relevant networks. The top four scoring networks were all cancer related: 1) proteoglycans in cancer (p-value = .0049), 2) Tumor necrosis factor (TNF) signaling pathway (p-value = .0053), 3) renal cell carcinoma (p-value = .0128), and 4) pathways in cancer (p-value = .0137). Importantly, these networks were all regulated by three miRNAs within the list of 12: 1) miR-652-5p, 2) miR-7-2-3p and 3) miR-520h. The three miRNAs were predicted to target four mutually shared genes: VHL, JUN, GAB1 and VEGFA, which are key players in the aforementioned pathways.

### Selection of miRNAs candidates associated with EAC metastasis

A panel of the top five candidate miRNAs related to disease progression were selected based on 1) regulation of the main pathways identified by DIANA-mirPath analysis (miR-652-5p, miR-7-2-3p, miR-520h), 2) downregulation in the metastatic group compared to Stage I Non-Progressors (miR-3925-3p), and 3) correlation with an EAC metastatic rat model in our recent study (miR-219b-3p) [19].

### Validation of selected miRNAs by qRT-PCR

Quantitative RT-PCR was performed, and four of the five candidate miRNAs showed significant downregulation in Stage I Progressors, compared to Stage I Non-Progressors. These included miR-652-5p (p-value = .045), miR-7-2-3p (p-value = .007), miR-3925-3p (p-value = .005), and miR-219b-3p (p-value = .003). MiR-520h did not show a significant difference between the two groups (p-value = .051) upon qRT-PCR validation, and it was excluded from further analysis (Figure 1).

**Figure 2:**
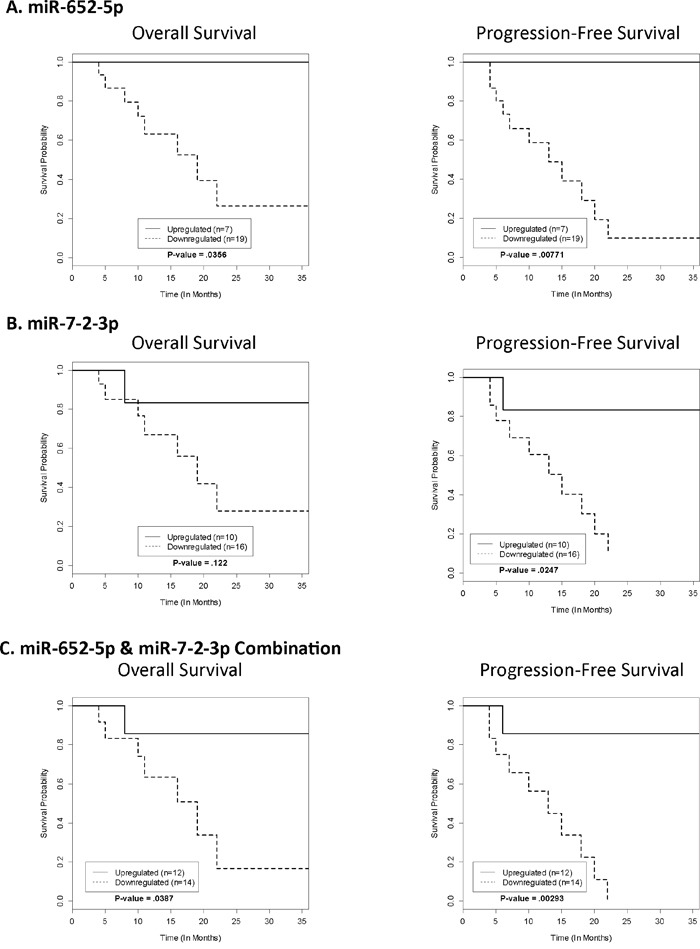
Kaplan-Meier survival analysis for OS and PFS The Kaplan-Meier Survival Curves for the testing set represent the OS and PFS of each significant miRNA based on their respective cut-off points generated from the training set.

### Correlation of selected miRNA expression with overall and progression-free survival

**Figure 3:**
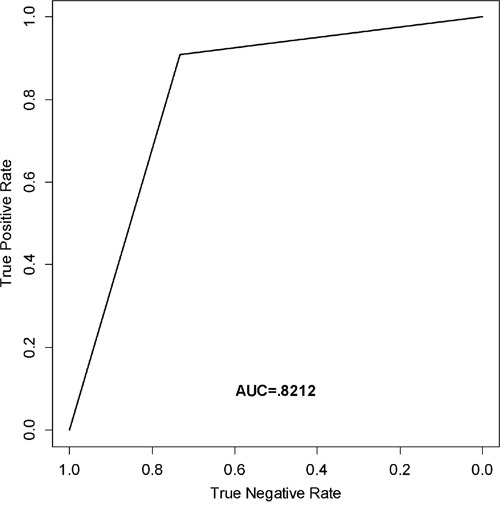
ROC curve analysis Receiver operating characteristic curve (ROC) generated from the testing set to estimate the classification performance of the model containing miR-652-5p and miR-7-2-3p.

The optimal cut-off point was developed to maximize sensitivity and specificity of classification (Progressors vs. Non-Progressors) for each miRNA. Each cut-off was developed based on the training set RQ values, independent of one another, and applied to the testing set. The RQ values that were used as a cut-off point were 14.00 for miR-652-5p, 5.00 for miR-7-2-3p, 1.00 for miR-3925-3p, and 25.00 for miR-219b-3p. There was a statistically significant difference in OS between Progressors and Non-Progressors for miR-652-5p (p-value = .0356) only. Whereas, for PFS there were statistically significant differences for miR-652-5p (p-value = .00771) and miR-7-2-3p (p-value = .0247) only. Therefore, a combination of miRNAs was created using those with statistically significant PFS (miR-652-5p and miR-7-2-3p). If a subject had an RQ value ≤ 14.00 for miR-652-5p AND an RQ value ≤ 5.00 for miR-3925-3p, it was considered a Progressor. If one or more of the values did not fall below the threshold, the subject was considered a Non-Progressor. OS (p-value = .0387) and PFS (p-value =.00293) for the multivariate model were both statistically significant. Survival curves for OS and PFS are shown in Figure 2, and the associated sensitivity, specificity, and accuracy (69, 73 and 81% for miR-652-5p, miR-7-2-3p, and the combination of miR-652-5p and miR-7-2-3p, respectively) are shown in Table 1. Overall, the Non-Progressors had a median survival advantage of at least 23, 21, and 23 months for miR-652-5p, miR-7-2-3p, and the combination of miR-652-5p and miR-7-2-3p when used as prognostic classifiers, respectively. Lastly, ROC analysis yielded the best class prediction accuracy (i.e., likelihood of progression) with an area under the curve (AUC) of .8212 for the combination of miR-652-5p and miR-7-2-3p (Figure 3).

**Table 1:** Classification performance

miRNA	Sensitivity	Specificity	Accuracy	Positive Predictive Value	Negative Predictive Value
**miR-652-5p**	100%	47%	69%	58%	100%
**miR-7-2-3p**	91%	60%	73%	63%	90%
**Combination of miR-652-5p & miR-7-2-3p**	91%	73%	81%	71%	92%

Classification performance for significant individual miRNAs in the testing set.

**Figure 4:**
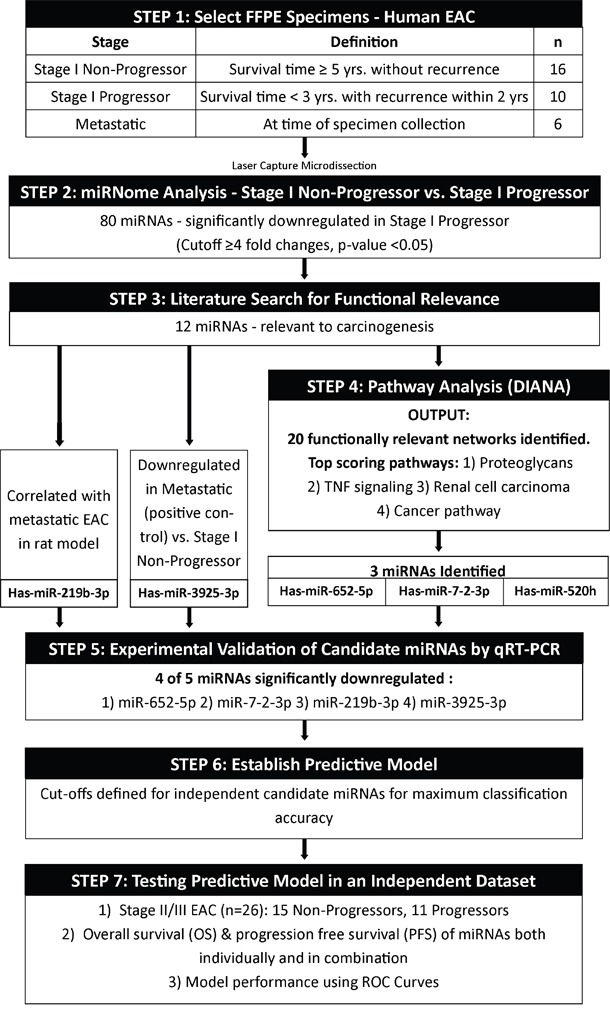
Study design Study schema representing the major steps in the experimental design.

## DISCUSSION

Early identification of EAC and a reliable prediction of prognosis are essential to the development of individualized therapeutic strategies, and there is increasing evidence that aberrant expressions of miRNAs are closely associated with disease progression and carcinogenesis [20–22]. Still, the prognostic significance of miRNAs in EAC has not been well-established. The present study demonstrates a discovery phase experiment that identifies and validates four miRNAs (miR-652-5p, miR-7-2-3p, miR-3925-3p, and miR-219-3p) that are significant and functionally relevant to EAC progression in Stage I EAC patients. In other words, these miRNAs can potentially be used to identify patients who are at high-risk of developing EAC recurrence following resection. We further evaluated the clinical relevance of the four miRNA signatures as a prognostic signature in an independent set of resectable stage II/III EAC patients. Our findings confirmed a novel panel of two miRNAs, miR-652-5p and miR-7-2-3p, that were significantly downregulated in correlation with diminished overall survival and progression-free survival. This dual panel could be used to ascertain tumor aggressiveness and guide therapeutic management.

The miRNA expression profiles associated with progression and metastasis of patients with EAC were explored using miRNA microarrays. The four miRNAs (miR-652-5p, miR-7-2-3p, miR-3925-3p, and miR-219-3p) that passed validation by qRT-PCR were consistent with several studies of other solid tumor types. Specifically, miR-7-2 and miR-219-3p were significantly downregulated in metastatic papillary thyroid carcinoma patients and metastatic gastric cancer progression patients, respectively [23, 24].

In order to more effectively characterize a specific signature for patients at high-risk for EAC progression, LCM was performed to isolate a highly-enriched pure cell population from the heterogeneous tissue section, and subsequently, miRNA microarray was performed [25, 26]. Although a number of previous studies utilized whole tumor tissues, the inclusion of non-tumor cells may inadvertently conceal the specific signature of the particular cell-type of interest. Wang et al. demonstrated the feasibility and potential power of discovering cell type-specific miRNA biomarkers in colorectal tumor tissue using the combination of LCM with genome-wide miRNA analysis [27]. Therefore, combination of LCM and high-throughput microarrays is an ideal method for cell type-specific miRNA expression profiling in solid tumors.

Multimodality therapy including the use of neoadjuvant chemoradiation with a platinum combined with either 5-fluorouracil (5-FU) or paclitaxel followed by surgical resection is widely accepted as the current standard of care for locally advanced esophageal cancer [28, 29]. In fact, the results of our study demonstrated that patients with downregulated miR-652-5p and/or miR-7-2-3p expression level have a significantly lower progression-free survival time. Consequently, patients with significantly downregulated miR-652-5p and miR-7-2-3p expression may benefit by the addition of adjuvant systemic therapy in order to more aggressively combat recurrence. Currently, the role of adjuvant chemotherapy is controversial in EAC. The National Comprehensive Cancer Network (NCCN) recommends adjuvant chemoradiation, or only adjuvant chemotherapy if induction radiation was administered, for patients who have had resection of adenocarcinoma with either residual disease, node-positive disease, or T2-T4a [30]. There is no data showing a benefit of adjuvant chemotherapy in patients who have been already treated with trimodality therapy. Moreover, miR-652-5p and miR-7-2-3p could be key therapeutic targets for patients with resectable EAC in an adjuvant therapeutic setting helping to identify high-risk patients.

Overall, the current study demonstrates significant evidence to suggest the clinical utility of the candidate miRNAs as prognostic markers for EAC progression and recurrence. The value may be emphasized by the discovery of a unique miRNA profile using the tissue obtained from endoscopic biopsy prior to therapy and resection. A future clinical trial to further evaluate the prognostic value of the miRNA panel may have the potential to enhance the clinical management of patients with EAC in several ways. First, it may help to stratify the multimodality therapy with regard to the severity of disease and effectiveness of therapy. Patients at high risk for progression and recurrence could receive more aggressive neoadjuvant management or the addition of adjuvant therapy to potentially improve survival. Conversely, patients at low-risk for progression may be able to avoid the toxicity from additional aggressive therapies. Second, it may help to individualize follow-up care, such as the frequency of surveillance imaging.

Major limitations of the study included a small sample size of the Stage I discovery set, and the testing set had an overall lack of survival information and did not reflect early stage disease. Many patients were not 3 years out from surgery and were therefore censored off during survival analysis. Additionally, it would be valuable to perform a prospective validation study with Stage I EAC samples to determine if the panel demonstrates utility in patients with early stage disease. Still, the clinical utility of the current study applies to the vast majority of patients presenting with locally advanced disease and at higher-risk for progression and recurrence. Lastly, there may be a minor node bias in the testing set, as many of those that were N2 and N3 at the time of surgery were also classified as Progressors by downregulation of miRNA. However, intuitively this would be expected because the biopsies were collected prior to neoadjuvant therapy and resection and stratified the patients that would have N2 or N3 disease according to RQ values. For future studies, a non-invasive validated test for these markers in blood may offer even broader clinical utility.

In conclusion, the present study identified a unique miRNA signature that was relevant to EAC progression and metastasis within the primary tumor, and subsequently, we demonstrated miR-652-5p/miR-7-2-3p signature might have clinical utility as a prognostic marker for surgically resectable EAC patients who could benefit from more aggressive adjuvant chemotherapy. This signature may be used to predict tumor response and disease progression, selection of therapeutic targets, and for personalized medicine approaches.

## MATERIALS AND METHODS

### Ethics statement

The study was performed under the approval of the Institutional Review Board at Allegheny Health Network (14-043: Molecular analysis across various stages of EAC progression). All patient samples were obtained with full written consent, and all samples were collected from tissues that remained after the completion of diagnosis from pathology.

### Study population and experimental design

The discovery set, referred herein as training set, constituted of 32 formalin-fixed paraffin-embedded (FFPE) samples: (1) Stage I Non-Progressor (n=16), survival time greater than 5 years without disease recurrence; (2) Stage I Progressor (n=10), survival time less than 3 years with recurrence within 2 years; and (3) Stage IV metastatic EAC (n=6). The three groups were classified based on TNM classification by the UICC-American Joint Committee on Cancer staging criteria (8th edition), survival time, and disease recurrence. Stage I Progressors and Stage I Non-Progressors were required to have a tumor size ≤ 3cm, node negativity, and tumor depth not involving the muscularis propria. Stage IV metastatic EAC samples were used as positive controls.

On all the training set samples, laser capture microdissection (LCM) was performed, and global miRNA profiles were generated. The analysis resulted in eighty significantly downregulated unique miRNAs in the Stage I Progressors compared to Stage I Non-Progressors. Subsequently, literature search based on functionality and relevance to cancer followed by pathway analysis provided a refined output of five candidate biomarkers. Quantitative reverse transcription-polymerase chain reaction (qRT-PCR) was performed to scientifically validate, and four miRNAs (miR-652-5p, miR-7-2-3p, miR-3925-3p, and miR-219b-3p) of the five candidate biomarkers were significantly different between groups.

As a next step, an independent cohort of FFPE samples, referred herein as testing set, was compromised of 26 patients with clinical and/or pathological stage II/III EAC. The patients did not have metastatic disease at the time of surgical intervention. Quantitative RT-PCR was performed on these samples for the four candidate miRNAs.

Next, the relative quantification (RQ) data from the training set was used to establish individual biomarker cutoffs and subsequently applied to the testing set to determine classification performance and survival benefit for the candidate miRNAs, defined as Progressors and Non-Progressors (Figure 4).

Histologic confirmation of EAC was performed by an experienced GI pathologist prior to LCM. Additionally, patient demographics and clinical data for both the training and testing sets are provided in Table 2.

**Table 2:** Clinical characteristics of study cohort

Charasteristic	Training Set		Testing Set	
	Non-Progressor	Progressor	Non-Progressor	Progressor
	n = 16	n = 10	n = 15	n = 11
**Age (Mean, Range)**	62 (49-80)	66 (52-84)	62 (48-76)	56 (34-81)
**Gender**				
Male (%)	13 (81%)	10 (100%)	15 (100%)	10 (91%)
Female (%)	3 (19%)		0 (0%)	1 (9%)
**Clinical Stage**				
0	2	0	0	0
I	10	10	0	0
II	1	0	4	4
III	0	0	11	7
Unknown	3	0	0	0
**Pathological Stage**				
0	0	1	8	0
I	13	4	4	2
II	0	0	1	2
III	0	0	2	7
Unknown	3	5	0	0
**Lymph node status**				
N0	16	10	13	4
N1	0	0	0	3
N2	0	0	2	3
N3	0	0	0	1

Patient demographics and clinical characteristics for both the training and testing sets.

### Laser capture microdissection

FFPE EAC tissues were laser-microdissected for molecular analysis. Briefly, 10μm tissue sections were placed onto irradiated polyethylene napthalate (PEN) membrane slides using a microtome. The resulting slides were incubated at 60°C for 2 hours in a dry oven to improve tissue adhesion to the membrane. Sections were stained with cresyl violet, and LCM was performed on all samples to collect tumor epithelial cells, using a LMD6500 Laser Microdissection system (Leica, Wetzlar, Germany). LCM sections were collected in 10 μL of deparaffinization solution (Qiagen, Valencia, CA; #19093) in irradiated 0.5 mL micro centrifuge tubes. To avoid RNA degradation, staining and LCM of each sample were completed within 30 minutes, and samples were immediately stored at -80°C.

### MiRNA profiling

MiRNA profiling was performed using miScript miRNA human miRNome (V21) PCR array (Qiagen, Valencia, CA) to evaluate the distinctive miRNA expression associated with EAC progression. Briefly, total RNA, containing miRNA, was isolated using the miRNeasy FFPE Kit (Qiagen, Valencia, CA; #217504) from post-LCM tissues, according to the manufacture's guidelines. RNA yield and purity (260/280 and 260/230 ratios) were determined using a NanoDrop spectrophotometer (Thermo Fisher Scientific, Waltham, MA). Next 50 ng of total RNA was reverse transcribed at 37°C for 1 hour, followed by inactivation of reverse transcriptase at 95°C for 5 minutes using miScript II RT kit (Qiagen, Valencia, CA; #218161). After the reverse transcription reactions were diluted 5-fold in nuclease-free water, a pre-amplification step was performed on all cDNAs using miScript PreAmp PCR Kit (Qiagen, Valencia, CA; #331452) with miScript PreAmp Primer mix (human miRNome (V21)). Cycling parameters for preamplification were: 95°C for 15 minutes, followed by 12 cycles of 94°C for 30 seconds, and 60°C for 3 minutes. After pre-amplified cDNA was diluted 20-fold in nuclease free water, pre-amplified cDNA samples were assayed using miScript miRNA human miRNome (V21) PCR array. PCR arrays were performed according to the manufacturer's instructions using miScript SYBR Green PCR Kit (Qiagen, Valencia, CA; #218073). Real-time PCR reactions were conducted at 95°C for 15 minutes, followed by 40 cycles of 94°C for 15 seconds, 55°C for 30 seconds, and 70°C for 30 seconds, using ABI 7900 SDS Real Time Instrument (Thermo Fisher Scientific, Waltham, MA). Relative gene expression was calculated using the ΔΔ-Ct method by QIAGEN's online data analysis tool, Gene Globe ((http://www.qiagen.com/us/shop/genes-and-pathways/data-analysis-center-overview-page/). SNORD95 and SNORD96A were selected as miRNA endogenous controls. Intergroup comparisons were performed, and the top differentially expressed miRNAs were identified using miScript miRNA Array Data Analysis (Qiagen, Valencia, CA), based on the following criteria: 1) greater than 4-fold change and 2) a p-value <0.05 for significance.

### Literature search for functional relevance

Literature search was performed on the global profiling data to identify the miRNAs with the highest relevance to cancer, particularly esophageal cancer. Candidate miRNAs with association and functional relevance to carcinogenesis were selected for further downstream analysis.

### Pathway analysis

The selected miRNAs from the literature search were analyzed via DIANA-mirPath analysis tool to determine a miRNA signature for EAC progression and metastasis [31]. DIANA predicted miRNA targets provided by the microT-CDS algorithm and experimentally validated miRNA interactions derived from DIANA-TarBase [32, 33, 34]. The graphical output of the program provided an overview of the genes of the pathway deregulated by candidate miRNAs. The statistical significance value associated with the identified biological pathways was calculated using mirPath.

### Validation of selected miRNAs by qRT-PCR

Quantitative RT-PCR was performed to scientifically validate the expression pattern of selected miRNAs using the same RNA samples used for the global miRNA profiling because qRT-PCR has been shown to provide greater sensitivity and higher specificity for measuring miRNA expression than microarray [35, 36]. A cohort of 15 Stage I Non-Progressor samples and 10 Stage I Progressor samples were used. One Stage I Non-Progressor sample was excluded from qRT-PCR validation analysis due to limited RNA yield. After total RNA was reversed transcribed using the RT^2^ First Strand Kit (Qiagen, Valencia, CA #330401), a pre-amplification step was performed as previously described. qRT-PCR was performed with the miScript miRNA SYBR Green PCR Kit (Qiagen, Valencia, CA #218075) in a total volume of 25 μl using the following RT^2^ Primer Assays: Hs_miR-520h_2 (Qiagen, Valencia, CA #MS44926), Hs_miR-652_5p_1 (Qiagen, Valencia, CA #MS37905), Hs_miR-7-2_1 (Qiagen, Valencia, CA #MS10542), Hs_miR-3925-3p_1 (Qiagen, Valencia, CA #MS41601), Hs_miR-2964a_3p_1 (Qiagen, Valencia, CA #MS42133) for each reaction on StepOnePlus real-time quantitative system (Applied Biosystems, Carlsbad, CA). Raw data was exported from the real-time instrument software and fold regulation was calculated using the ΔΔ-Ct method. SNORD95 and miR-16 were selected as miRNA endogenous controls. Intergroup comparisons were performed and normalized by the pathologically confirmed LCM normal esophagus sample.

### Data analysis and survival analysis

All statistical analyses were conducted using SPSS software (IBM, Armonk, NY, Version 23) and R (R Foundation for Statistical Computing, Vienna, Austria). An independent t-test was used to identify a difference between miRNA levels between Progressors and Non-Progressors in the training set. Due to the small sample size and a right skew in the boxplots in Figure 1, a nonparametric Mann-Whitney U-test was used to confirm findings from the independent t-test. P-values of <0.05 were considered to be statistically significant.

An independent testing set was assembled to evaluate the prognostic power of the candidate miRNAs in terms of overall survival (OS) and progression-free survival (PFS), and corroborate clinical utility of the candidate miRNAs. OS was defined as the time interval from the date of surgical resection to the date of cancer-related death or loss to follow-up. PFS was defined as the time interval from the date of surgical intervention to the date of tumor recurrence/metastasis or loss to follow-up. This testing set of 26 patients with stage II/III disease did not have metastatic disease at the time of surgical intervention. A patient was considered a Progressor if he/she developed metastatic disease within 3 years.

Each of the candidate miRNA's (miR-652-5p, miR-7-2-3p, miR-3925-3p, and miR-219b-3p) expression was measured in the testing set using qRT-PCR from FFPE specimens as described above.

Receiver operating characteristic (ROC) curve analysis was used to establish the threshold for each miRNA, to define the cut-off of whether a subject showed downregulated (Progressor) or upregulated (Non-Progressor) miRNA expression [37]. A Log-Rank Test and Kaplan-Meier Survival Curves in R were used in the testing set to investigate if there was a difference in OS and PFS between patients classified as upregulated or downregulated (A Package for Survival Analysis in S. Version 2.38).

## References

[1] Siegel RL, Miller KD, Jemal A (2015). Cancer statistics, 2015. CA Cancer J Clin.

[2] DeMeester SR (2006). Adenocarcinoma of the esophagus and cardia: a review of the disease and its treatment. Ann Surg Oncol.

[3] Pohl H, Sirovich B, Welch HG (2010). Esophageal adenocarcinoma incidence: are we reaching the peak?. Cancer Epidemiol Biomarkers Prev.

[4] Lee JH (2005). Interventional gastroenterology: esophageal and pancreatic cancers. Seminars in oncology.

[5] Zhang Y (2013). Epidemiology of esophageal cancer. World J Gastroenterol.

[6] Rubenstein JH, Chen JW (2014). Epidemiology of gastroesophageal reflux disease. Gastroenterol Clin North Am.

[7] Talsma K, van Hagen P, Grotenhuis BA, Steyerberg EW, Tilanus HW, van Lanschot JJ, Wijnhoven BP (2012). Comparison of the 6th and 7th Editions of the UICC-AJCC TNM Classification for Esophageal Cancer. Ann Surg Oncol.

[8] Yates LA, Norbury CJ, Gilbert RJ (2013). The long and short of microRNA. Cell.

[9] Bartel DP (2004). MicroRNAs: genomics, biogenesis, mechanism, and function. Cell.

[10] Jansson MD, Lund AH (2012). MicroRNA and cancer. Mol Oncol.

[11] Schetter AJ, Leung SY, Sohn JJ, Zanetti KA, Bowman ED, Yanaihara N, Yuen ST, Chan TL, Kwong DL, Au GK, Liu CG, Calin GA, Croce CM, Harris CC (2008). MicroRNA expression profiles associated with prognosis and therapeutic outcome in colon adenocarcinoma. JAMA.

[12] Iorio MV, Ferracin M, Liu CG, Veronese A, Spizzo R, Sabbioni S, Magri E, Pedriali M, Fabbri M, Campiglio M, Menard S, Palazzo JP, Rosenberg A, Musiani P, Volinia S, Nenci I (2005). MicroRNA gene expression deregulation in human breast cancer. Cancer Res.

[13] Croce CM (2009). Causes and consequences of microRNA dysregulation in cancer. Nat Rev Genet.

[14] Hiyoshi Y, Kamohara H, Karashima R, Sato N, Imamura Y, Nagai Y, Yoshida N, Toyama E, Hayashi N, Watanabe M, Baba H (2009). MicroRNA-21 regulates the proliferation and invasion in esophageal squamous cell carcinoma. Clinical cancer research.

[15] Liu R, Liao J, Yang M, Sheng J, Yang H, Wang Y, Pan E, Guo W, Pu Y, Kim SJ, Yin L (2012). The cluster of miR-143 and miR-145 affects the risk for esophageal squamous cell carcinoma through co-regulating fascin homolog 1. PloS one.

[16] Feber A, Xi L, Luketich JD, Pennathur A, Landreneau RJ, Wu M, Swanson SJ, Godfrey TE, Litle VR (2008). MicroRNA expression profiles of esophageal cancer. J Thorac Cardiovasc Surg.

[17] Garman KS, Owzar K, Hauser ER, Westfall K, Anderson BR, Souza RF, Diehl AM, Provenzale D, Shaheen NJ (2013). MicroRNA expression differentiates squamous epithelium from Barrett's esophagus and esophageal cancer. Dig Dis Sci.

[18] Yang H, Gu J, Wang KK, Zhang W, Xing J, Chen Z, Ajani JA, Wu X (2009). MicroRNA expression signatures in Barrett's esophagus and esophageal adenocarcinoma. Clin Cancer Res.

[19] Zaidi AH, Kelly LA, Kreft RE, Barlek M, Omstead AN, Matsui D, Boyd NH, Gazarik KE, Heit MI, Nistico L, Kasi PM, Spirk TL, Byers B, Lloyd EJ, Landreneau RJ, Jobe BA (2015). Associations of microbiota and toll-like receptor signaling pathway in esophageal adenocarcinoma. BMC cancer.

[20] Gao G, Gay HA, Chernock RD, Zhang TR, Luo J, Thorstad WL, Lewis JS, Wang X (2013). A microRNA expression signature for the prognosis of oropharyngeal squamous cell carcinoma. Cancer.

[21] Liu N, Chen NY, Cui RX, Li WF, Li Y, Wei RR, Zhang MY, Sun Y, Huang BJ, Chen M, He QM, Jiang N, Chen L, Cho WC, Yun JP, Zeng J (2012). Prognostic value of a microRNA signature in nasopharyngeal carcinoma: a microRNA expression analysis. Lancet Oncol.

[22] Vosgha H, Salajegheh A, Smith RA, Lam AK (2014). The important roles of miR-205 in normal physiology, cancers and as a potential therapeutic target. Curr Cancer Drug Targets.

[23] Ab Mutalib NS, Othman SN, Mohamad Yusof A, Abdullah Suhaimi SN, Muhammad R, Jamal R (2016). Integrated microRNA, gene expression and transcription factors signature in papillary thyroid cancer with lymph node metastasis. PeerJ.

[24] Lei H, Zou D, Li Z, Luo M, Dong L, Wang B, Yin H, Ma Y, Liu C, Wang F, Zhang J, Yu J, Li Y (2013). MicroRNA-219-2-3p functions as a tumor suppressor in gastric cancer and is regulated by DNA methylation. PLoS One.

[25] Bonner RF, Emmert-Buck M, Cole K, Pohida T, Chuaqui R, Goldstein S, Liotta LA (1997). Laser capture microdissection: molecular analysis of tissue. Science.

[26] Emmert-Buck MR, Bonner RF, Smith PD, Chuaqui RF, Zhuang Z, Goldstein SR, Weiss RA, Liotta LA (1996). Laser capture microdissection. Science.

[27] Wang S, Wang L, Zhu T, Gao X, Li J, Wu Y, Zhu H (2010). Improvement of tissue preparation for laser capture microdissection: application for cell type-specific miRNA expression profiling in colorectal tumors. BMC Genomics.

[28] Sjoquist KM, Burmeister BH, Smithers BM, Zalcberg JR, Simes RJ, Barbour A, Gebski V, Australasian Gastro-Intestinal Trials G (2011). Survival after neoadjuvant chemotherapy or chemoradiotherapy for resectable oesophageal carcinoma: an updated meta-analysis. Lancet Oncol.

[29] van Hagen P, Hulshof MC, van Lanschot JJ, Steyerberg EW, van Berge Henegouwen MI, Wijnhoven BP, Richel DJ, Nieuwenhuijzen GA, Hospers GA, Bonenkamp JJ, Cuesta MA, Blaisse RJ, Busch OR, ten Kate FJ, Creemers GJ, Punt CJ (2012). Preoperative chemoradiotherapy for esophageal or junctional cancer. N Engl J Med.

[30] Ajani JA, Barthel JS, Bentrem DJ, D'Amico TA, Das P, Denlinger CS, Fuchs CS, Gerdes H, Glasgow RE, Hayman JA, Hofstetter WL, Ilson DH, Keswani RN, Kleinberg LR, Korn WM, Lockhart AC (2011). Esophageal and esophagogastric junction cancers. Journal of the National Comprehensive Cancer Network.

[31] Papadopoulos GL, Alexiou P, Maragkakis M, Reczko M, Hatzigeorgiou AG (2009). DIANA-mirPath: Integrating human and mouse microRNAs in pathways. Bioinformatics.

[32] Reczko M, Maragkakis M, Alexiou P, Grosse I, Hatzigeorgiou AG (2012). Functional microRNA targets in protein coding sequences. Bioinformatics.

[33] Paraskevopoulou MD, Georgakilas G, Kostoulas N, Vlachos IS, Vergoulis T, Reczko M, Filippidis C, Dalamagas T, Hatzigeorgiou AG (2013). DIANA-microT web server v5. 0: service integration into miRNA functional analysis workflows. Nucleic Acids Res.

[34] Hsu SD, Lin FM, Wu WY, Liang C, Huang WC, Chan WL, Tsai WT, Chen GZ, Lee CJ, Chiu CM, Chien CH, Wu MC, Huang CY, Tsou AP, Huang HD (2011). miRTarBase: a database curates experimentally validated microRNA-target interactions. Nucleic Acids Res.

[35] Chen Y, Gelfond JA, McManus LM, Shireman PK (2009). Reproducibility of quantitative RT-PCR array in miRNA expression profiling and comparison with microarray analysis. BMC Genomics.

[36] Koshiol J, Wang E, Zhao Y, Marincola F, Landi MT (2010). Strengths and limitations of laboratory procedures for microRNA detection. Cancer Epidemiol Biomarkers Prev.

[37] Robin X, Turck N, Hainard A, Tiberti N, Lisacek F, Sanchez JC, Muller M (2011). pROC: an open-source package for R and S+ to analyze and compare ROC curves. BMC Bioinformatics.

